# Cisplatin causes erectile dysfunction by decreasing endothelial and smooth muscle content and inducing cavernosal nerve senescence in rats

**DOI:** 10.3389/fendo.2023.1096723

**Published:** 2023-01-25

**Authors:** Yinghao Yin, Yihong Zhou, Jun Zhou, Liangyu Zhao, Hongji Hu, Ming Xiao, Bin Niu, Jingxuan Peng, Yingbo Dai, Yuxin Tang

**Affiliations:** ^1^ Department of Urology, The Fifth Affiliated Hospital, Sun Yat-sen University, Zhuhai, Guangdong, China; ^2^ Guangdong Provincial Key Laboratory of Biomedical Imaging, The Fifth Affiliated Hospital, Sun Yat-sen University, Zhuhai, Guangdong, China; ^3^ Department of Urology, First Affiliated Hospital of Jishou University, Jishou, Hunan, China

**Keywords:** cisplatin, erectile dysfunction, apoptosis, senescence, cancer survivor

## Abstract

**Introduction:**

Cisplatin (cis‐diamminedichloroplatinum II, CDDP), a drug widely used for cancer worldwide, may affect erectile function, but its side effects have not received enough attention. To investigate the effect of CDDP on erectile function and its possible mechanism.

**Methods:**

Sprague−Dawley rats were intraperitoneally administered CDDP (CDDP group) or the same volume of normal saline (control group). Erectile function was evaluated after a one-week washout. Then, histologic changes in the corpus cavernosum and cavernous nerve (CN) were measured. Other Sprague-Dawley rats were used to isolate the major pelvic ganglion and cavernous nerve (MPG/CN). RSC96 cells were then treated with CDDP. SA-β-gal staining was used to identify senescent cells, and qPCR was used to detect the senescence-associated secretory phenotype (SASP). Finally, the supernatant of RSC96 cells was used to culture MPG/CN. Erectile function was measured after administration of CDDP. The cavernosum levels of α-SMA, CD31, eNOS, and γ-H2AX, the apoptosis rate and the expression of p16, p21 and p53 in CN were also assayed. The senescent phenotype of RSC96 cells treated with CDDP was identified, and neurite growth from the MPG/CN was photographed and measured.

**Results:**

The CDDP group had a significantly lower ICP/MAP ratio than the control group. Compared to the control group, the CDDP group exhibited significantly lower α-SMA, CD31 and eNOS levels and significantly higher γ-H2AX and apoptosis rates in corpus cavernosum. In addition, CDDP increased some senescence markers p16, p21 and p53 in CN. *In vitro*, CDDP induced RSC96 senescence and SASP, and the supernatant of senescent cells slowed neurite outgrowth of MPG/CN.

**Discussions:**

CDDP treatment could induce erectile dysfunction, by affecting the content of endothelial and smooth muscle and causing SASP in CN. The results indicate that CDDP treatment should be considered as a risk factor for ED. Clinicians should pay more attention to the erectile function of cancer patients who receive CDDP treatment.

## Introduction

In 2020, there were 19.3 million new cases of cancer and 10.0 million cancer deaths worldwide. The global cancer burden is expected to rise 47% from 2020, with 28.4 million cases in 2040 (1). Given the increasing incidence and prevalence of cancer, chemotherapy is a well-established treatment strategy. The role of chemotherapy will continue to expand and play a more important role in improving both the survival and quality of life of patients. CDDP is a widely used drug to treat various solid cancers such as testicular (2), ovarian (3), head and neck (4), bladder (5), lung (6), cervical cancer (7), lymphomas (8) and several others (9). However, the adverse effects of CDDP limit its effectiveness and become an inherent challenge for its application.

Erectile dysfunction is not usually mentioned as a side effect. However, 42% of colorectal cancer patients who were treated with oxaliplatin had erectile problem (10). Tomoya Kataoka et al. reported that oxaliplatin causes erectile dysfunction in rats due to endothelial dysfunction (11), providing further confirmation that platinum-based drugs can indeed induce ED and that this problem is often overlooked by clinicians. CDDP, a main chemotherapy agent for testicular tumors, has been reported to be associated with erectile dysfunction (12, 13). Moreover, testicular tumors are the most common tumors in young men. Therefore, it is necessary to clarify the relationship between CDDP and erectile function.

In this study, we studied erectile function in rats after administration of CDDP. Illustrating the mechanism, underlying the effect of CDDP on erectile function, will help doctors take measures to protect erectile function in cancer survivors.

## Materials and methods

### Cells and drugs

RSC96, the Schwann cell line of the rat sciatic nerve, was purchased from Procell (Wuhan, China). Briefly, cells were maintained in DMEM (Gibco Life Technologies, CA, USA) supplemented with 10% fetal bovine serum (Gibco Life Technologies, CA, USA) and 1% penicillin-streptomycin (Gibco Life Technologies, CA, USA) at 37 ˚C in a humidified atmosphere of 5% CO_2._ CDDP was acquired from APExBIO (Houston, USA) and dissolved in normal saline.

### Cell viability assay (MTT)

Cell viability was evaluated at different time points (48 hours, 72 hours) using the 3-(4,5-dimethylthiazol-2-yl)-2,5-dip-henyltetrazolium bromide (MTT) assay. RSC96 cells were seeded in 96-well plates at a density of 3×10^3^ cells/well and cultured for 24 hours. Then, the cells were cotreated with different concentrations of CDDP in the medium. At different time points, 10 μL of MTT was added to each well and incubated at 37°C for an additional 4 hours. Then, the supernatant was carefully removed, and dimethyl sulfoxide was added to each well to dissolve the crystals by gentle agitation for 10 minutes. For each well, the absorbance at 490 nm was estimated on a microplate reader (Bio-Tek, VT, USA).

### SA-β-gal staining

Cells were seeded in 6-well plates at a density of approximately 20 000 cells/well for 24 hours, and then treated with different concentrations of CDDP for 2 days. Thereafter, the medium was removed and replaced with complete medium (without CDDP) for another 2 days. Then, the cells were fixed and stained using a Senescence β-Galactosidase Staining Kit (Solarbio Life Science, Beijing, China).

### RNA extraction and quantitative reverse transcription-PCR

For qPCR, total RNA was extracted using the RNA Kit (Omega Bio-Tek, USA) according to the manufacturer’s instructions. Reverse transcription was performed using a HiScript II 1^st^ strand cDNA synthesis kit (Vazyme, China). qPCR was performed on a step-one plus real-time PCR system (Bio-Rad, CA, USA) using ChamQ universal SYBR qPCR master mix (Vazyme, China). All primers were synthesized by Ribobio Co., Ltd. (Guang Zhou, China). Primer sequences are listed in **Table 1**. β-actin was used as an internal control. The relative levels of RNAs were calculated using the 2-ΔΔCt method.

**Table 1:** Primers for qPCR.

Gene symbol	5’-3’
IL6-F	GTCAACTCCATCTGCCCTTCAG
IL6-R	GGCAGTGGCTGTCAACAACAT
TGFB1-F	GCGCCTGCAGAGATTCAAGTCAAC
TGFB1-R	GTATCAGTGGGGGTCAGCAGCC
β-actin -F	TCAGGTCATCACTATCGGCAAT
β-actin -R	AAAGAAAGGGTGTAAAACGCA
TGFB2 -F	TGCTGAGAACCTTTTTGCTCC
TGFB2-R	GTCGAGGGTGCTGCAGGTA
ICAM-1-F	GTCGGTGCTCAGGTATCCATC
ICAM-1 -R	TCGTCTTTCATCCAGTTAGTCTCC
IL1A -F	AAATACTCAGCTCTTTGTGAGTGC
IL1A -R	TGTGATGAGTTTTGGTGTTTCC
CCL2 -F	CTCTTCCTCCACCACTATGC
CCL2 -R	CTCTGTCATACTGGTCACTTC
CTGF -F	CAGGCTGGAGAAGCAGAGTCGT
CTGF -R	CTGGTGCAGCCAGAAAGCTCAA
PAI1 -F	CCATCTCCGTGCCCATGAT
PAI1 -R	GTCATGTTGCTCTTCCATTGTCT

### Major pelvic ganglion and cavernous nerve culture

Bilateral MPG/CN (entire MPG with a 2-mm length CN attached) complexes from each rat were isolated and excised intact. Each MPG/CN complex was placed in a 6-well plate and then covered with 50 μL of Matrigel (Corning, NY, USA) as previously described (14). After incubation at 37 °C for 5 minutes, 2.0 mL of complete culture medium and 2.0 ml supernatant from RSC96 treated with or without CDDP were added according to the grouping. RSC96 cells were treated with CDDP for 2 days, and the medium was removed and replaced with complete culture medium (without CDDP) for another 2 days. The supernatant was then used to culture the MPG/CN complex.

### Animal treatment

**Figure 1:**
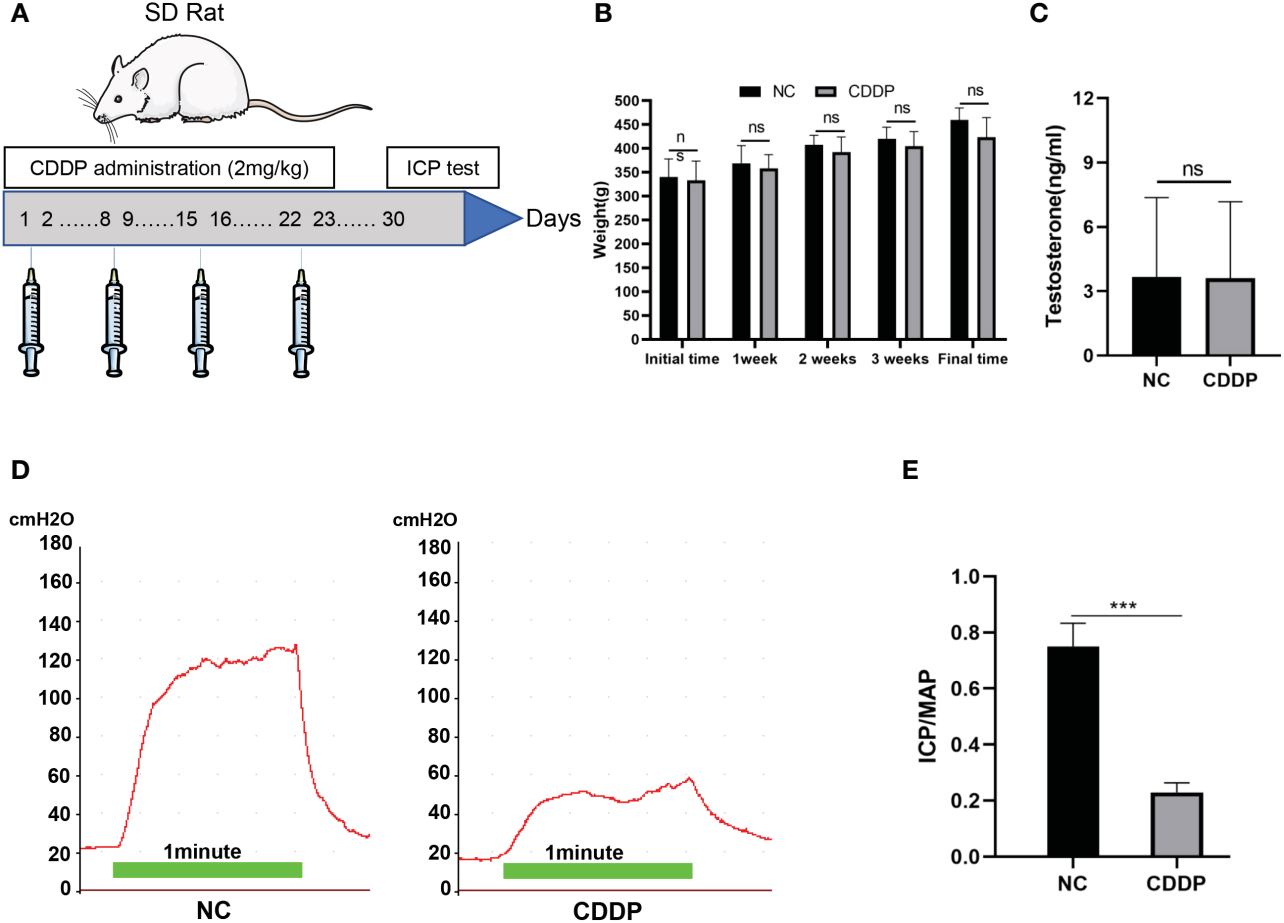
Characterization of the CIED rat model. **(A)** Experimental scheme for the development of the CIED rat model. CDDP was administered i.p. twice a week for 4 weeks. After a week washout, animals were evaluated to assess erectile function. **(B)** Changes in rat body weight over time. Initial weight levels in the 2 groups of rats after 7 days of adaptive feeding; final weight after 7 days of washout at 4 weeks. **(C)** Testosterone levels of each group were shown. **(D)** Representative images of ICP in response to electrical stimulation of the cavernosal nerve; **(E)** Results of the ratio of MaxICP to MAP in each group. ns: not significant, ***P < 0.001.

All animal experiments in this study were approved by the Institutional Animal Care and Use Committee of the Fifth Affiliated Hospital of Sun Yat-sen University.Twenty 12-week-old male Sprague−Dawley rats were used for this study. The rats were divided into 2 groups: The CDDP group rats (n = 10) were intraperitoneally administered 2 mg/kg body weight of CDDP on Days 1, 2, 8, 9, 15, 16, 22, and 23 (**Figure 1A**). And the others (n=10) injected with normal saline as the control group. The dose was based on some scholars’ recommendation about the dose translation from animals to humans (15, 16). The erectile function was evaluated after a one-week washout.

### Measurement of erectile function

Intracavernous pressure (ICP) was measured by electrical stimulation, as previously reported (17, 18). All rats were anesthetized by injection with pentobarbital (40 mg/kg). Then, the carotid artery was exposed and cannulated with a PE-50 tube which was connected to a pressure transducer to continuously monitor arterial pressure. A 25-gauge needle containing 100 IU/mL heparin solution was inserted into the right penile crura and the other end of the tube was also connected to a pressure transducer to monitor intracavernous pressure (ICP). The cavernous nerve was electrically stimulated at 1 V, 20 Hz, with a pulse width of 5 ms for 1 minute and a 5-minute interval before subsequent stimulation. The ratios of maximal ICP (MaxICP) to mean arterial pressure (MAP) were calculated to evaluate erectile function *in vivo*.

### Measurement of serum concentration of testosterone

After the ICP test was completed, clinical needles and vacuum tubes were used to collect blood through the inferior vena cava.The serum was separated immediately and stored at 80°C. The levels of testosterone were assessed using ELISA kits from Solarbio (Beijing, China).

### Histologic examinations

#### H&E staining

The staining procedures were reported in our previous study (18, 19). Slides containing tissue sections were deparaffinized using a dry oven at 60°C for 30 minutes and in 2 changes of xylene for 10 minutes, and then rehydrated in a series of decreasing concentrations of alcohol. Finally, the slides were washed in tap water. After that, Harris’s hematoxylin reagent was used to stain for 8 minutes. The slides were then destained in 0.5% acid-alcohol for 3 seconds and washed with running water. The slides were counterstained with 0.5% eosin for another 1 minute. After dehydration with different concentrations of alcohol and xylene, the slides were observed with a microscope for histopathologic examination.

#### Masson trichrome staining

The staining procedures were reported in our previous study (18). Tissue sections of the middle part of the penis were stained according to Masson kit instructions (Solarbio, Beijing, China). The collagen tissues (stained blue) of the penile sponge and smooth muscle tissues (stained red) were observed under a light microscope. The cavernous smooth muscle/collagen ratio was analyzed using ImageJ software.

#### Immunofluorescence

Slides containing tissue sections were deparaffinized with xylene and rehydrated with ethanol. Then, antigen retrieval was performed by placing the slides in Tris/EDTA buffer (Solarbio Life Science, Beijing, China) before heating for 10 minutes. The slides were then treated with endogenous peroxidase blocker for 10 minutes and normal 10% goat serum (Solarbio Life Science, Beijing, China) was utilized for 30 minutes to block nonspecific binding sites. Different antibodies were applied to the slides and incubated overnight at 4 °C. After the hybridization of secondary antibodies, DAPI was used to stain the cell nucleus. The slides were observed using a fluorescence microscope (OLYMPUS, Tokyo, Japan). Antibodies against γ-H2AX, p16, p21 and p53 were obtained from Abclonal Biotechnology (Wuhan, China); CD31 antibody was purchased from Bioworld Technology (Nanjing, China); eNOS antibody was obtained from Abcam Biotechnology (Cambridge, UK). α-SMA antibody was purchased from Affinity (OH, USA).

#### TUNEL staining

TUNEL staining was carried out according to previous studies (18). Apoptosis of the corpus cavernosum of each group was detected using a TUNEL apoptosis detection kit (KeyGEN BioTECH, Nanjing, China) following the manufacturer’s instructions. Each sample was randomly selected from 4 fields of view. Cells with green staining were counted as apoptotic, and the ratio of apoptotic cells to the total number of cells in the field of view yielded the rate of apoptosis.

### Statistical analysis

Results were analyzed using GraphPad Prism 8.0 (GraphPad Software, San Diego, CA, USA) and expressed as the mean ± standard deviation (SD). Statistical analyses were performed using the two-tailed Student’s t test. Differences among groups were considered significant at a P value less than 0.05.

## Results

### CDDP weakens the erectile function of rats

As shown in **Figures 1B, C**, the mean body weight and testosterone levels did not differ significantly between the two groups, but erectile function had obvious difference (**Figures 1D, E**). Erectile function was assessed by MaxICP and MaxICP/MAP. The results showed that both MaxICP and MaxICP/MAP revealed a significant decrease in the CDDP group compared to control rats.

### Effects of CDDP on smooth muscle contents and endothelium function in corpus cavernosum

**Figure 2:**
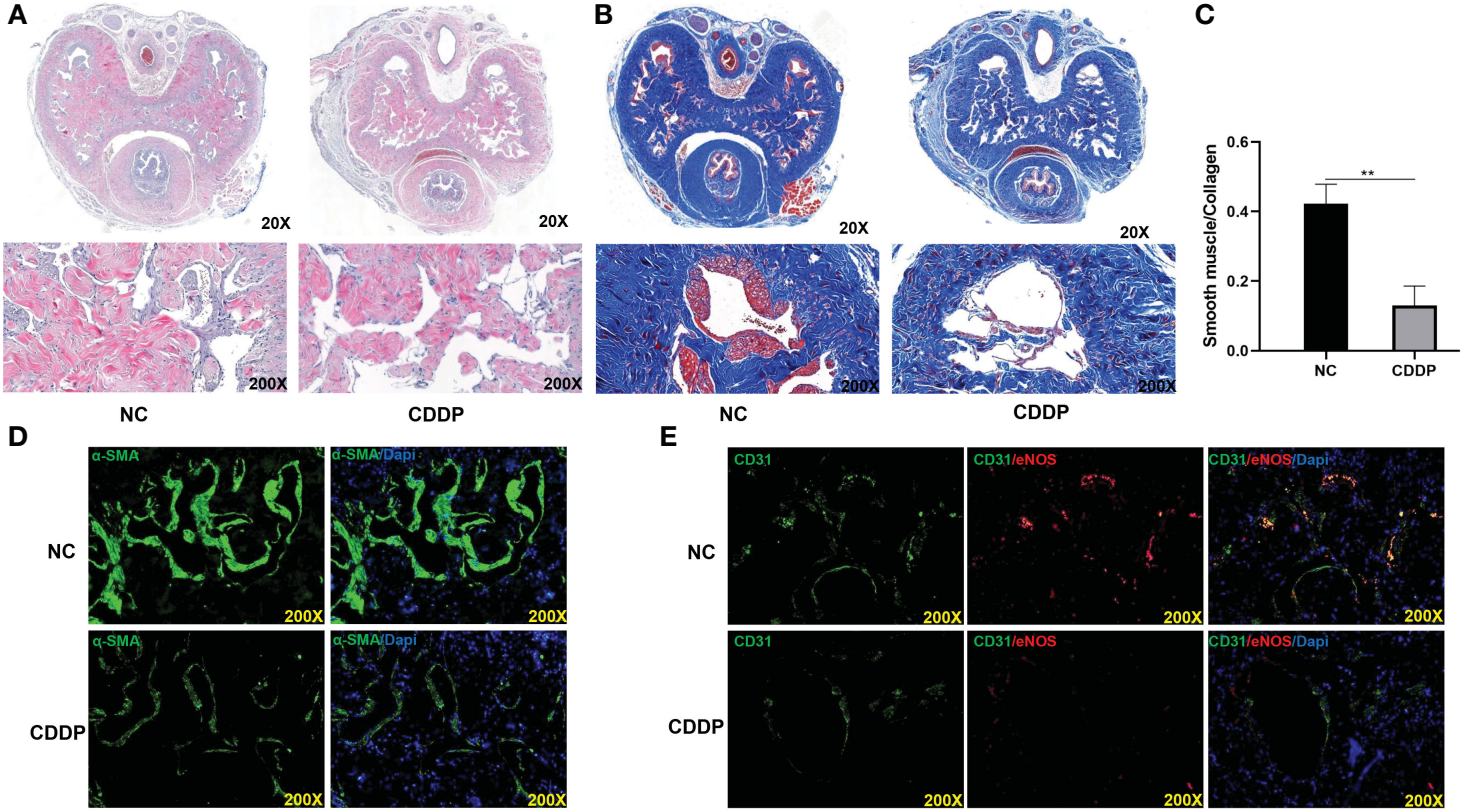
Effect of CDDP on smooth muscle and endothelium contents in the corpus cavernosum **(A)** H&E staining. A thinner smooth muscle layer was found in all CDDP-treated rats; **(B)** Masson trichrome staining. Smooth muscle manifested as red, and connective tissue was blue. **(C)** The ratio of smooth muscle to collagen. **(D)** Representative images of immunofluorescence staining of α-SMA in each group. **(E)** Representative images of immunofluorescence staining of CD31(green) and eNOS (red) in the corpus cavernosum from age-matched control and CDDP-treated rats. **P < 0.01.

The morphological changes and smooth muscle (SM)-to-collagen ratios of the different groups were detected with H&E and Masson’s trichrome staining, as shown in **Figures 2A**–**C**. CDDP significantly caused structural disorders and decreased smooth muscle mass in the corpus cavernosum. Moreover, Immunofluorescence staining with an anti-α-SMA antibody showed a significant reduction in smooth muscle content in CDDP-treated rats compared with normal control rats (**Figure 2D**). Cavernosal endothelial dysfunction is recognized as a hallmark of the disease pathology. Tomoya Kataoka found that oxaliplatin causes erectile dysfunction in rats due to endothelial dysfunction (11). Consistent with the findings, we found the endothelial cell content and eNOS was severely decreased in the CDDP group (**Figure 2E**).

### CDDP induces DNA damage and apoptosis in corpus cavernosum

**Figure 3:**
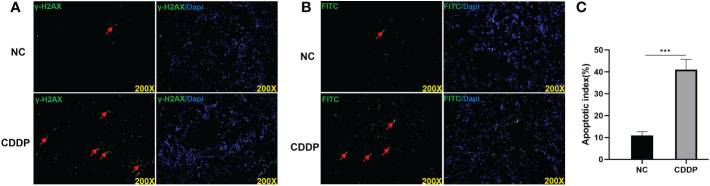
CDDP increased DNA damage and apoptosis in the corpus cavernosum. **(A)** Representative images of immunofluorescence staining of γ-H2AX, a DNA damage marker, in the corpus cavernosum from age-matched control and CDDP-treated rats. **(B)** Representative images of TUNEL staining in the corpus cavernosum from age matched control and CDDP-treated rats; **(C)** The apoptotic index, the percentage of apoptotic cells (stained green) of all cells, to quantify the cavernous apoptosis level. Red arrows showed the positive signals, ***P < 0.001.

CDDP binds to DNA to cause a biological effect by forming CDDP-DNA adducts and induceing DNA damage response. Phosphorylated H2AX (γ-H2AX) is known to be a marker to investigate the effects on DNA damage (20). As shown in **Figure 3A**, CDDP treatment significantly increased γ-H2AX levels compared with the control group in the corpus cavernosum. The apoptotic pathway may be triggered in cells after CDDP treatment. We measured the apoptosis level in corpus cavernosum by TUNEL (**Figures 3B, C**). In the CDDP group, there were a dramatically larger percentage of apoptosis cells than the control group. The aforementioned results indicates that CDDP can induces DNA damage and apoptosis in corpus cavernosum.

### CDDP causes senescence in CN

**Figure 4:**
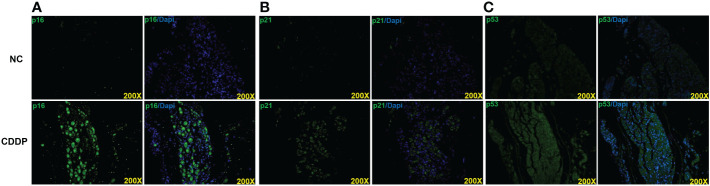
CDDP induced senescence in CN. Representative images of immunofluorescence staining of p16 **(A)**, p21 **(B)** and p53 **(C)** in each group.

ED is considered a complication of CDDP-induced peripheral neuropathy (12). Aina Calls found that CDDP-induced peripheral neuropathy was associated with neuronal senescence-like response (21). In senescent cells, the levels of cell cycle inhibitors, including p16, p21, and p53 were augmented. Following CDDP treatment, the expression levels of the p16, p21, and p53 were increased in CN (**Figure 4**).

### CDDP induces Senescence and SASP in RSC96 cells

**Figure 5:**
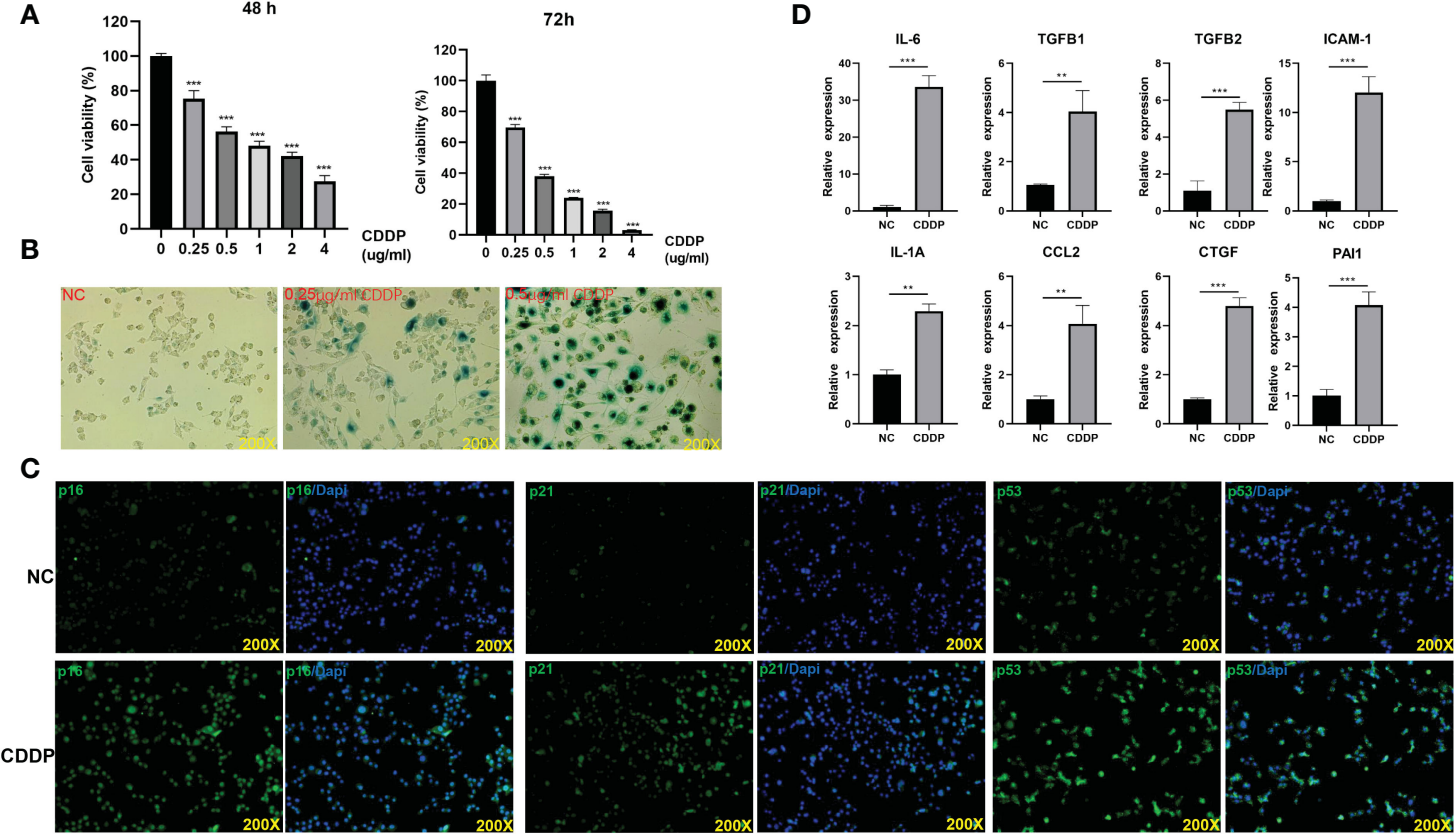
CDDP could induce senescence in RSC96 cells. **(A)** The effect of different concentrations of CDDP on the viability of RSC96 cells was detected by MTT at 48 and 72 hours. **(B)** RSC96 cells were treated with the indicated concentrations and then stained for SA-β-gal activity at 96 hours. **(C)** After RSC96 cells were treated with CDDP (0.5 μg/ml), immunofluorescence staining was performed to detect the expression of p53, p21 and p16 in RSC96 cells. **(D)** qPCR was used to detect the SASP-related genes after RSC96 cells were treated with CDDP (0.5 μg/ml). **P < 0.01, ***P < 0.001.

To determine RSC96 cells response to CDDP, an MTT assay was performed. Notably, the proliferation viability of cells decreased with increasing concentration (**Figure 5A**). The SA-β-gal staining assay confirmed our hypothesis that senescence occurred in RSC96 cells treated with CDDP (**Figure 5B**). Immunofluorescence illustrated that CDDP upregulated the expression of senescence-related genes: p16, p21 and p53 (**Figure 5C**). SASP is another typical feature of senescent cells. CDDP promoted the expression of SASP-related genes, IL-6, TGFβ1, ICAM1, TGFβ2, CCL2, IL-1α and CXCL1 (**Figure 5D**).

### Senescent Schwann cells affects the function of CN

**Figure 6:**
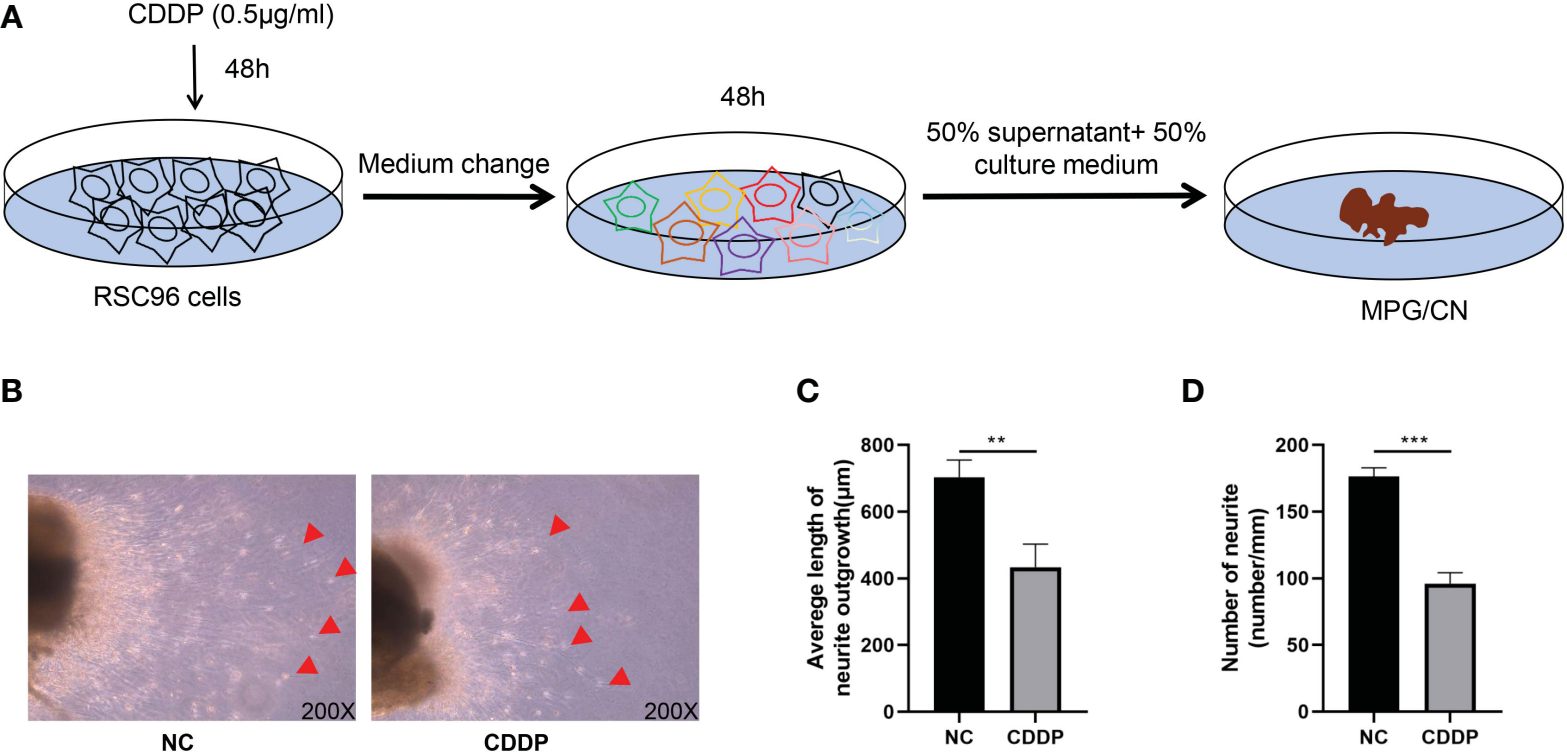
Senescent Schwann cells reduced neurite outgrowth from CN *in vitro*. **(A)** A schematic diagram of supernatant isolation and MPG treated with supernatant. **(B)** Neurite outgrowth from CN 120 hours after seeding. Original magnification x200. The red arrow indicates outgrowing fibers, **(C)**. The supernatant of senescent cells inhibited fiber outgrowth from CN. **(D)**. The supernatant of senescent cells decreased the number of fibers from CN. **P < 0.01, ***P < 0.001.

To investigate the effect of senescent Schwann cells on axonal growth of the CN, we used an in vitro model of MPG/CN culture (14). As shown in **Figure 6A**, the supernatant of RSC9 cells was used to treat the MPG/CN system. We dissected the MPG with 2 mm of CN attached and cultured the tissue in vitro. After 120 hours, new neurite outgrowths from the end of the CN were measured. CDDP significantly restrained the neurite outgrowth of CN (**Figures 6B–D**).

## Discussion

Many anticancer agents used in cancer chemotherapy possess either cytotoxic or cytocidal activity. However, they can injure both the cancer cells and the normal tissue and cells of patients because of their nonselectivity. The dysfunctions of normal cells and organs caused by these agents are called side effects. A variety of complications that cause suffering and lower quality of life make it harder for doctors. Many side effects have been reported and could be overcome. However, there are still problems to be resolved (22). Several studies have demonstrated that platinum-based chemotherapy can induce ED (10, 12, 13, 23, 24), but ED has not gained the attention of clinicians as a complication. Some scholars have found that oxaliplatin can induce ED in rats by inducing damage to the endothelium of the corpus cavernosum (11). CDDP is a conventional chemotherapeutic agent for numerous tumors. In this study, administration of CDDP to rats resulted in a decrease in the MaxICP/MAP ratio, endothelium and smooth muscle content and eNOS level. In addition, CDDP induces senescence in Schwann cells, which triggers the SASP phenotype, thereby affecting CN function. Therefore, CDDP is confirmed to be able to induce ED by affecting the corpus cavernosum and CN. CDDP exposure can cause testicular damage and a significant reduction in testosterone level (25, 26). In our study, the CDDP-treated rats showed no different serum testosterone levels than the control group, which might be caused by the use of a lower concentration of CDDP. eNOS has an indispensable role in the erectile response (27) and endothelial dysfunction is common in ED induced by diabetes mellitus (28), bilateral cavernous nerve injury (29) and oxaliplatin (11). Long-term cardiovascular events have often been reported to increase in patients with platinum-based chemotherapy due to vascular toxicity and endothelial dysfunction. In our results, CD31 staining and eNOS levels in the corpus cavernosum were decreased after CDDP treatment. CDDP can induce apoptosis in HUVECs (30). Furthermore, CDDP also upregulates NF-κB/ICAM-1 to affect the production of nitric oxide (NO) and cGMP, which leads to endothelial dysfunction (31). Moreover, CDDP also caused loss of smooth muscle. CDDP exerts anticancer activity *via* multiple mechanisms but the generation of DNA lesions is the most acceptable mechanism, followed by activation of the apoptosis pathway (9, 32). Correspondingly, our results showed that γ-H2AX, a DNA damage marker, and TUNEL staining were augmented in corpus cavernosum of CDDP group. Hence, we hypothesized that CDDP might damage DNA and cause endothelial and smooth muscle cell apoptosis, which impairs the erectile function of rats. Of course, the specific mechanism by which CDDP affects the endothelial and smooth muscle cells of the corpus cavernosum deserves further study.

Peripheral neurotoxicity is a common side effect of platinum-based chemotherapy (33). Some researchers have even considered ED as a neuropathic subscale-related symptom (10). A recent study found that CDDP-induced peripheral neuropathy is associated with neuronal senescence-like response (21). In our results, the expression of senescence-related markers was up-regulated in CN after CDDP treatment. Schwann cells help nerve cells to transmit information faster by wrapping their long extensions in myelin. Abnormal production of myelin can perturb signal transmission between nerve cells, which leads to neurological defects (34). Schwann cells in the peripheral nervous system play a pivotal role in nerve repair (35). In addition, dysfunction of Schwann cells plays an important role in the pathogenesis of diabetic peripheral neuropathy (36, 37). *In vitro*, we found that CDDP was indeed able to induce senescence in RSC96 cells and generate SASP. Inflammation is a key driver of pathological changes in many peripheral neuropathy (38). We used the supernatant of RSC96 cells to culture MPG/CN and indeed found that the growth of CN decelerated in the CDDP group, which indirectly indicated that the substances secreted by senescent Schwann cells could affect the function of CN.

In summary, our findings indicate that the CDDP-induced reduction in endothelial and smooth muscle content and SASP, which leads to CN dysfunction, are responsible for ED in rats.

In this study, we used normal rats administered CDDP, and the absence of cancer model experiments was one limitation of the present study. In addition, the effects of different dose and frequency of administration of CDDP on erectile function should be evaluated in the future. Another limitation of this study is that the direct effects of CDDP on nerves were not investigated. The underlying mechanism of effects of CDDP on CN needs to be further explored.

## Conclusion

Our study revealed that CDDP treatment could induce erectile dysfunction by affecting the content of endothelial and smooth muscle and causing SASP in CN. Thus, erectile function in cancer survivors receiving CDDP chemotherapy should receive the attention of clinicians.

## Data availability statement

The original contributions presented in the study are included in the article/supplementary material. Further inquiries can be directed to the corresponding authors.

## Ethics statement

The animal study was reviewed and approved by the Ethics Committee of the Fifth Affiliated Hospital, Sun Yat-sen University.

## Author contributions

Conceptualization, YY, YD and YT; Data curation, YY, JZ, LZ and YD, Formal analysis, YY, JZ, LZ, BN and JP, Funding acquisition, LZ, YZ and YT, Methodology, YY, HH, MX, and JP, Project administration, YD and YT, Supervision, YD and YT, Validation, YY, BN and YZ; Writing – Original Draft, YY, YD and YT, Writing – Review & Editing, YY, YD and YT. All authors contributed to the article and approved the submitted version.
